# An umbrella review of physical-activity therapy and cognitive behavioral therapy in reducing fear of falling among community-dwelling older adults: insights on intervention intensity and duration

**DOI:** 10.3389/fpubh.2024.1498451

**Published:** 2025-01-03

**Authors:** Yuan Sheng, Caili Wang, Yan Wang, LunPing Pan, Mengmeng Zhang, Deshan Liu, Wei Gao

**Affiliations:** ^1^School of Nursing and Rehabilitation, Shandong University, Jinan, China; ^2^Department of Traditional Chinese Medicine, Qilu Hospital of Shandong University, Jinan, China; ^3^Department of PICC Clinic, Qilu Hospital of Shandong University, Jinan, China

**Keywords:** fear of falling, aged, physical-activity therapy, cognitive behavioral therapy, umbrella review

## Abstract

**Introduction:**

Data about the impact of varying physical-activity therapy (PAT) intensities and the ideal duration of cognitive behavioral therapy (CBT) on older adults is inadequate. In this umbrella review, we seek to comprehensively synthesize and analyze findings from systematic reviews and meta-analyses regarding the optimal PAT intensity for lowering FOF and the duration of CBT interventions for effectively lowering FOF.

**Methods:**

The PubMed, Web of Science, Cochrane Library, Medline, Embase, and CINAHL databases were searched up to April 2024. AMSTAR 2 was applied to assess the methodological and reporting quality. The quality of evidence for each intervention’s effect was evaluated using GRADE guidelines. A further meta-analysis of the primary studies was conducted to evaluate the effects of PAT intensity and CBT duration.

**Results:**

In the 18 included studies, 12 were PAT interventions, 3 were multifactorial, and 3 were CBT interventions. The umbrella review found that PAT and CBT interventions can effectively manage FOF. Comparable improvements were reported with low- and moderate-intensity PAT intervention (*p* < 0.0001); Significant improvements were observed with CBT immediately post-intervention, in the short-term (<6 months), and in the long-term (≥6 months) (*p* < 0.0001).

**Discussion:**

Our study revealed that a comprehensive intervention strategy combining low or moderate PAT with CBT interventions is more effective than isolated approaches, as it addresses the multifaceted nature of fear and fall risk. Future research should continue to track the long-term effects of synergistic interventions to optimize fall prevention strategies for older populations.

**Systematic review registration:**

https://www.crd.york.ac.uk/prospero Identifier CRD42024557893.

## 1 Introduction

The phenomenon of falls among older adults is a significant public health concern, particularly as the global population ages. A large population-based study revealed that approximately 27.5% of 65 and older experience falls annually, increasing to 32.8–35.7% in individuals aged 85 and older (1). Su et al. (2) further demonstrated that the fall risk among individuals over 75 years was three times higher compared to those aged 65 to 74, and this risk escalated to nine times higher for individuals over 95 years. The accumulation of risk factors, including muscle weakness, balance deficits, and cognitive decline, is critical in fall susceptibility. For instance, Gale et al. (3) highlighted that intrinsic factors, such as muscle weakness and gait disorders, are prevalent among older adults and play a significant role in fall risk. Similarly, Nguyen et al. (4) found that body composition, interconnected with age, gender, health perception, and financial status, exacerbates the risk of falls.

While age is a significant fall risk factor, it is not the sole determinant. Environmental factors also play a critical role in fall risk among older adults. A review has shown that simple changes, such as improving lighting, removing clutter, and installing grab bars, can significantly reduce fall risk in hospital and home settings (5). The integration of wearable technologies into these modifications, such as smart belts equipped with airbag systems, has been developed to mitigate the impact of falls (6). Mobile applications also significantly advance fall risk management, especially in the COVID-19 pandemic, where physical distancing has become essential (7). Furthermore, Şimşek et al. (8) suggested that factors like living alone and fear of falling (FOF) are important contributors to fall risk. FOF is defined as low perceived self-efficacy in preventing falls during daily activities, with a prevalence of about 49.60%, ranging from 6.96 to 90.34% (9, 10).

FOF can increase fall risk in various ways. Firstly, older adults who experience FOF may avoid activities that they perceive as risky, leading to decreased muscle strength and impaired balance, both of which are significant predictors of falls. For instance, a study showed that older adults with limitations in lower limb exercises exhibited a markedly higher FOF, contributing to decreased physical performance (11). This avoidance behavior creates a vicious cycle in which FOF leads to inactivity, further weakening physical capabilities and increasing fall risk (12). In addition to physical factors, psychological conditions, such as anxiety and depression, are commonly associated with FOF, further heightening fall risk. For instance, Young et al. (13) noted that distressing thoughts can reduce the effectiveness of the reach-and-grasp balance response prior to a balance perturbation, thereby increasing falls. Similarly, Lin et al. (14) emphasized that emotional distress not only affects mental well-being but also negatively impacts physical health by mediating resilience.

The relationship between FOF and fall risk is characterized by a complex interplay of physical and psychological factors, and understanding these dynamics is crucial for developing effective prevention strategies. Physical-activity therapy (PAT), such as structured exercise programs, has been shown to improve balance, strength, and overall physical fitness, thereby reducing FOF. A recent study found that older adults who participated in structured exercise programs experienced significant reductions in FOF, particularly those who began with higher baseline fears (15). Moreover, eHealth-delivered exercise programs that combine PAT with educational components have proven particularly effective (16). Besides, Cognitive Behavioral Therapy (CBT) has shown promise in reducing FOF by addressing cognitive distortions and maladaptive beliefs that contribute to fear. A systematic review concluded that CBT-based interventions significantly reduce FOF among community-dwelling older adults, suggesting that psychological interventions can enhance self-efficacy and coping strategies (17).

In summary, PAT and CBT are essential components of a holistic approach to managing FOF in older adults. By addressing the fear’s physical and psychological factors, these interventions can significantly improve the quality of life and functional independence of older adult individuals. However, considering the previous reviews/meta-analysis, we raise two questions: (1) What intensity of PAT is most suitable for older adults? (2) Does CBT have a long-term effect on older adult FOF? Therefore, this overview was carried out to provide a comprehensive review of PAT and CBT interventions, evaluate which PAT intensity is most helpful for lowering FOF and determine the effective duration of CBT for reducing FOF in older adults.

## 2 Materials and methods

### 2.1 Design

This umbrella review of systematic reviews and meta-analyses adheres to the Preferred Reporting Items for Overviews of Reviews (PRIOR) guidelines (18). The review protocol was registered in the PROSPERO International Prospective Register of Systematic Reviews (Ref: CRD42024557893).

### 2.2 Criteria for considering reviews for inclusion

The inclusion and exclusion criteria were defined using the PICOS framework. The inclusion criteria were as follows: (1) P (population): Participants in the included reviews/meta-analyses were community-dwelling older adults who had a mean age of ≥60 years. (2) I (intervention): Reviews/meta-analyses that include CBT or any form of PAT, either as a primary or secondary intervention, were considered. (3) C (comparator): no intervention, standard care, or wait-list control. (4) O (outcomes): Included studies were required to utilize at least one measure related to FOF, such as the Falls Efficacy Scale (FES), FES-International (FES-I), Short FES-I, Activities-Specific Balance Confidence Scale (ABC), Survey of Activities and Fear of Falling in the older adult (SAFE), Geriatric Fear of Falling Measure (GFFM), Fear of Falling Questionnaire (FFQ), and a measure of balance confidence (CONFbal). (5) S (study design): Only published systematic reviews or meta-analyses were considered. The exclusion criteria were: (1) Literature written in languages other than English. (2) Studies with primary or secondary outcomes unrelated to FoF (e.g., fall self-efficacy, balance confidence).

### 2.3 Search methods for identification of reviews

Searches were conducted until April 2024 on PubMed, Web of Science, The Cochrane Library, Medline, Embase, and CINAHL. The Reference lists of relevant reviews and included studies were manually checked for additional citations not captured in the initial search. A combination of text words and MeSH phrases, such as “aged/older adult/older/senior,” “fear of falling/concern about falling/afraid of falling/worry about falling,” and “meta-analysis/systematic review” were employed.

### 2.4 Data extraction and management

Two independent reviewers screened the titles and abstracts derived from the database for eligibility; subsequently, all potentially includable articles were stored in Endnote X9 software, and duplicates were removed. For final inclusion, two researchers separately evaluated the full texts of the selected articles to ensure they met the eligibility criteria. In cases of disagreement, a third researcher participated in reaching a consensus. The author, year, country, study design, participants, number of studies, interventions, quality assessment, and principal conclusions were extracted from the included literature.

### 2.5 Assessment of methodological quality of included reviews

Two authors independently evaluated the quality of the included reviews using AMSTAR 2 (A Measurement Tool to Assess Systematic Reviews 2) (19). It contains 16 items, of which seven are critical domains (items 2, 4, 7, 9, 11, 13, and 15). Review methodological quality is classified into four levels: high, moderate, low, and critically low. The two researchers double-checked their evaluations after finishing the quality assessment. When there were disagreements, a third researcher helped to resolve the inconsistency.

### 2.6 Assessment of evidence quality of included reviews

Two authors independently evaluated the quality of the evidence using GRADE (Grades of Recommendations Assessment, Development, and Evaluation) guidelines (20). Evidence quality was assessed based on limitations in design, inconsistency, indirectness, imprecision, and publication bias. The evidence was subsequently rated as high, moderate, low, or very low. Finally, the two researchers cross-checked their evaluations of quality. In cases of discrepancies, a third researcher participated to reach a consensus.

### 2.7 Data synthesis

This study involved an umbrella review and a supplementary meta-analysis. Initially, the outcomes of PAT and CBT interventions were qualitatively summarized. Data were then taken from the meta-analyses to elucidate the effects of these interventions. Direct comparisons between CBT and PAT interventions were unfeasible due to insufficient data. Subsequently, a meta-analysis was conducted utilizing Review Manager 5.4.1 to determine which PAT intensity is most helpful for lowering FOF and explore the effects of CBT interventions’ duration on reducing FOF in older adults. Also, heterogeneity was quantified by *I*^2^, with a value exceeding 50% indicating substantial heterogeneity. During the sensitivity analysis, papers of low quality were removed, and the combined effect was calculated as the standardized mean difference (SMD) with a 95% confidence interval. Publication bias was evaluated using funnel plots if more than 10 original papers were incorporated.

## 3 Results

### 3.1 Search results

**Figure 1:**
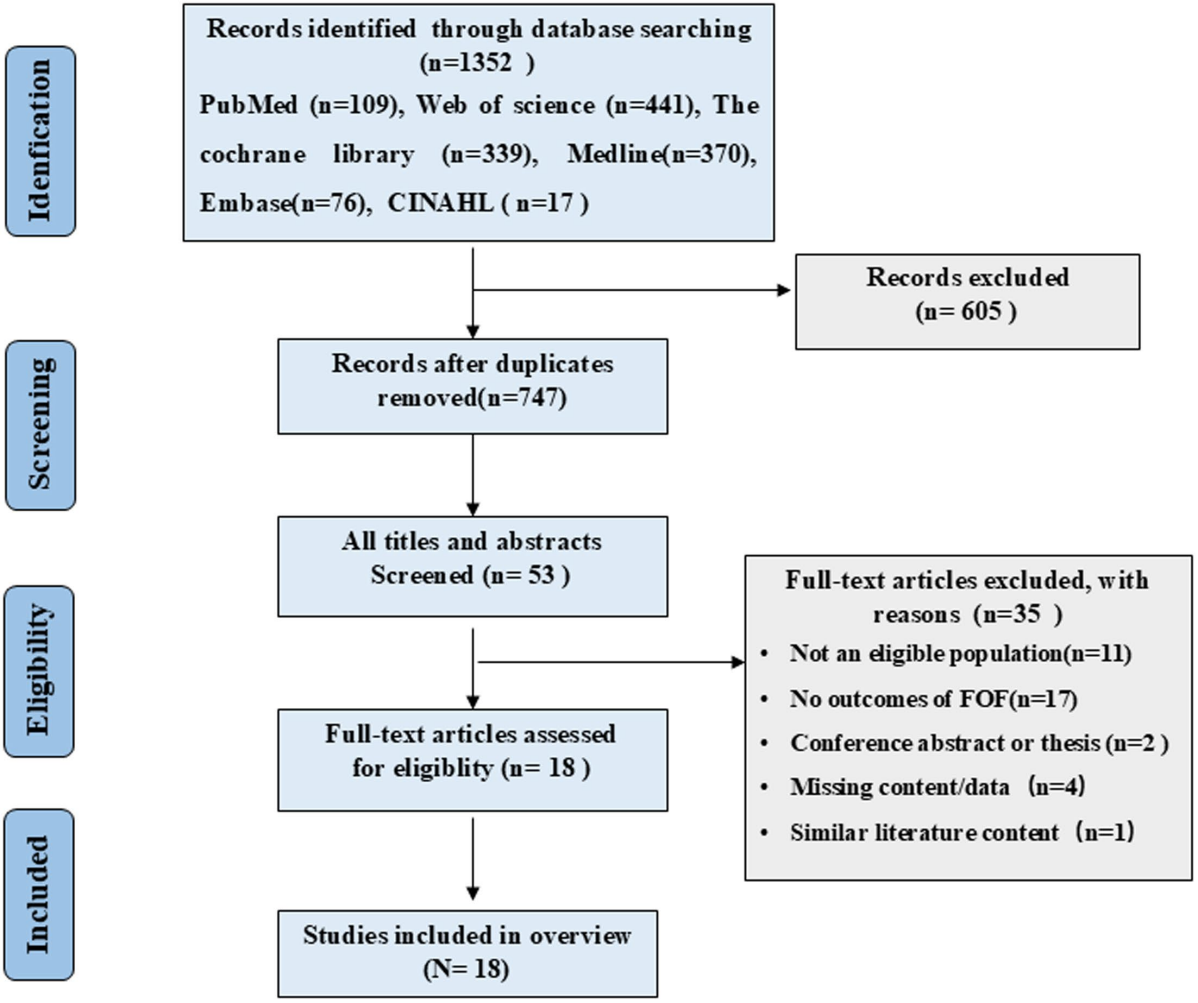
Flow chart of the study selection process.

For our overview of reviews, we retrieved 1,352 papers, of which 747 remained after duplicates were removed. After browsing the titles and abstracts, 53 potentially relevant papers were identified. After reading the complete text, we removed 35 papers, leaving 18 reviews/meta-analyses (Figure 1).

### 3.2 General characteristics and outcomes of included studies

In the 18 included studies, three studies (21–23) reported multi-component interventions targeting FOF in community-dwelling older adults, while the remaining studies focused on single interventions, including PAT (*n* = 12) (24–35) and CBT (*n* = 3) (17, 36, 37). The main characteristics of the reviewed studies are summarized in Table 1.

**Table 1:** General characteristics and outcomes of included reviews.

Author, year	Study design	Participants	Interventions	Quality assessment	Main conclusion
Racey et al., 2021 (21)	RCT/CCT	Community-dwelling adults (aged 50+)	No limitations	The Cochrane Handbook	PAT interventions probably reduce FOF to a small to moderate degree immediately post-intervention in community-living older people.
Kruisbrink et al., 2022 (22)	RCT	Older community-dwelling people	No limitations	The Cochrane Handbook	Interventions with meditation, holistic exercises (such as Tai Chi), or body awareness are more effective than interventions without these components.
Zijlstra et al., 2007 (23)	RCT	Community-living older people with a mean age of 65 and older	No limitations	The Cochrane Handbook	Home-based exercise, fall-related multifactorial programs and community-based tai chi delivered in group format have been effective in reducing FoF in community-living older people.
Chua et al., 2019 (17)	RCT	Community-dwelling people aged 60 years and older	CBT	The Cochrane Handbook	CBT-based multi-component interventions are effective at reducing FOF among community-dwelling older people.
Papadimitriou et al., 2020 (36)	RCT	Community people aged over 65 years	CBT	The Cochrane Handbook	CBT interventions were effective for FOF in both short-term (2–5 months) and long-term follow-ups (4–12 months).
Liu et al., 2018 (37)	RCT	Community-dwelling older people (≥60)	CBT	The Cochrane Handbook	CBT interventions have significant immediate and retention effects for up to 12 months on reducing FOF.
Savvakis et al., 2024 (24)	RCT	Aged 60 years and older	PAT	The Cochrane Handbook	PAT interventions could improve FoF among frail and pre-frail older people.
Kendrick et al., 2014 (25)	RCT/Quasi-RCT	Older people living in the community	PAT	The Cochrane Handbook	PAT interventions in community-dwelling older people probably reduce FOF to a limited extent immediately after the intervention.
Kumar et al., 2016 (26)	RCT/quasi-RCT	Older people living in the community	PAT	The Cochrane Handbook	PAT interventions probably reduce FOF to a small to moderate degree immediately post-intervention in community-living older people.
Feng et al., 2022 (27)	RCT	Community-dwelling older people	PAT	PEDro scale	In older people living in the community, PAT interventions had a small-to-moderate effect size on FOF.
Weber et al., 2020 (28)	RCT	Aged≥59 years	MBI, including Tai Chi, Qigong, Yoga, or Pilates	PEDro scale	MBI involving meditative movements may serve as a promising opportunity to improve FoF in older people.
Logghe et al., 2010 (29)	RCT	aged 50 years or older	Tai Chi	Delphi criteria list	There is insufficient evidence to conclude whether Tai Chi is effective in decreasing FOF over the age of 50 years.
Silva et al., 2021 (30)	RCT	Healthy older people	Pilates	PEDro Scale	Older people who attend Pilates reduce their FoF.
Veronese et al., 2017 (31)	RCT	Older people	Dance movement or therapy	None	No firm conclusions can be drawn.
Silva et al., 2018 (32)	RCT/ quasi-RCT	Aged 65 or older	Technology-mediated dance interventions	The Cochrane Handbook	The effectiveness of technology-mediated dance interventions on FOF in older people is weak.
Ge et al., 2022 (33)	RCT/Quasi-RCT/single group pre-post	Community-residing older adult	Exergames	Modified PeDro scale	Exergames may have a positive effect in reducing FOF in community-dwelling older people.
Melo et al., 2023 (34)	RCT/quasi-RCT	Healthy community-dwelling older people	The aquatic physical therapy exercises	The Cochrane Handbook	Compared with land-based physical therapy exercises, aquatic therapy exercises reduce FOF more effectively.
Papalia et al., 2020 (35)	RCT	Patients aged 65 or older	Land-based or aquatic exercise	The Cochrane Handbook	Physical exercise is not an effective treatment to reduce FOF in the older adult.

RCT, randomized controlled trial; CCT, clinical control trial; CBT, cognitive behavior therapy; PAT, physical-activity therapy; Mind–body interventions; AMOB/VLL, A Matter of Balance/Volunteer Lay Leader model.

### 3.3 Quality of included reviews

The methodological quality of the 18 included studies was evaluated using AMSTAR 2. Of these, one study was rated as high quality (25), one as moderate quality (27), four as low quality (21–23, 37), and 12 as critically low quality (17, 24, 26, 28–36). The AMSTAR 2 assessment for these multiple-system reviews is presented in Table 2.

**Table 2:** Assessment list of multiple system reviews (AMSTAR) 2 (*n* = 20).

Study	1	2^a^	3	4 ^a^	5	6	7^a^	8	9 ^a^	10	11^a^	12	13^a^	14	15 ^a^	16	Quality rating
Racey et al., 2021 (21)	Yes	Yes	Yes	Yes	Yes	Yes	No	Yes	Yes	No	Yes	Yes	Yes	Yes	Yes	Yes	Low
Kruisbrink et al., 2022 (22)	Yes	Yes	Yes	Yes	Yes	Yes	No	Yes	Yes	No	Yes	Yes	Yes	No	Yes	Yes	Low
Zijlstra et al., 2007 (23)	Yes	No	Yes	Yes	Yes	Yes	Yes	Yes	Yes	No	No meta-analysis	No meta-analysis	Yes	Yes	No meta-analysis	Yes	Low
Chua et al., 2019 (17)	Yes	No	Yes	Yes	No	No	No	Partial Yes	Yes	No	Yes	No	No	No	No	Yes	Critically low
Papadimitriou et al., 2020 (36)	Yes	Yes	Yes	Yes	Yes	Yes	No	Yes	Yes	No	No	No	No	No	No	Yes	Critically low
Liu et al., 2018 (37)	Yes	Yes	Yes	Yes	Yes	Yes	No	Yes	Yes	No	Yes	Yes	Yes	Yes	Yes	Yes	Low
Savvakis et al., 2024 (24)	Yes	No	Yes	Partial yes	Yes	Yes	No	Yes	Yes	No	No meta-analysis	No meta-analysis	Yes	Yes	No meta-analysis	Yes	Critically low
Kendrick et al., 2014 (25)	Yes	Yes	Yes	Yes	Yes	Yes	Yes	Yes	Yes	Yes	Yes	Yes	Yes	Yes	Yes	Yes	High
Kumar et al., 2016 (26)	Yes	No	Yes	Partial Yes	Yes	Yes	No	Partial Yes	Yes	No	Yes	Yes	Yes	Yes	Yes	Yes	Critically low
Feng et al., 2022 (27)	Yes	Yes	Yes	Yes	Yes	Yes	Yes	Yes	Yes	No	Yes	Yes	Yes	Yes	Yes	Yes	Moderate
Weber et al., 2020 (28)	Yes	No	Yes	Partial Yes	Yes	Yes	No	Yes	Yes	No	No	Yes	Yes	Yes	Yes	Yes	Critically low
Logghe et al., 2010 (29)	Yes	No	Yes	Partial Yes	Yes	Yes	No	No	Yes	No	Yes	No	No	Yes	No	Yes	Critically low
Silva et al., 2021 (30)	Yes	Yes	Yes	Partial Yes	Yes	Yes	No	Yes	Yes	No	Yes	Yes	No	No	No	Yes	Critically low
Veronese et al., 2017 (31)	No	Partial Yes	Yes	Yes	Yes	Yes	No	Yes	No	No	No meta-analysis	No meta-analysis	No	Yes	No meta-analysis	Yes	Critically low
Silva et al., 2018 (32)	Yes	Yes	Yes	Yes	Yes	Yes	No	Yes	Yes	No	Yes	Yes	Yes	Yes	No	Yes	Critically low
Ge et al., 2022 (33)	Yes	No	Yes	Partial Yes	Yes	Yes	No	Yes	Yes	No	No meta-analysis	No meta-analysis	No	Yes	No meta-analysis	Yes	Critically low
Melo et al., 2023 (34)	Yes	Yes	Yes	Partial Yes	Yes	Yes	No	Yes	Yes	No	No meta-analysis	No meta-analysis	Yes	Yes	No meta-analysis	Yes	Critically low
Papalia et al., 2020 (35)	Yes	No	Yes	Partial Yes	Yes	Yes	No	Yes	Yes	No	Yes	Yes	Yes	Yes	No	Yes	Critically low

a Critical items.

(1) Did the research questions and inclusion criteria for the review include the components of PICO? (2) Id the report of the review contain an explicit statement that the review methods were established prior to the conduct of the review and did the report justify any significant deviations from the protocol? (3) Did the review authors explain their selection of the study designs for inclusion in the review? (4) Did the review authors use a comprehensive literature search strategy? (5) Did the review authors perform study selection in duplicate? (6) Did the review authors perform data extraction in duplicate? (7) Did the review authors provide a list of excluded studies and justify the exclusions? (8) Did the review authors describe the included studies in adequate detail? (9) Did the review authors use a satisfactory technique for assessing the risk of bias (RoB) in individual studies that were included in the review? (10) Did the review authors report on the sources of funding for the studies included in the review? (11) If meta-analysis was performed did the review authors use appropriate methods for statistical combination of results? (12) If meta-analysis was performed, did the review authors assess the potential impact of RoB in individual studies on the results of the meta-analysis or other evidence synthesis? (13) Did the review authors account for RoB in individual studies when interpreting/discussing the results of the review? (14) Did the review authors provide a satisfactory explanation for, and discussion of, any heterogeneity observed in the results of the review? (15) If they performed quantitative synthesis did the review authors carry out an adequate investigation of publication bias (small study bias) and discuss its likely impact on the results of the review? (16) Did the review authors report any potential sources of conflict of interest, including any funding they received for conducting the review?

### 3.4 Quality of evidence

The quality of 31 pieces of evidence derived from the 18 included studies was evaluated using the GRADE system. Two pieces of evidence were rated as high quality, eight as moderate quality, 12 as low quality, and nine as very low quality. Table 3 displays the quality grade for intervention outcomes.

**Table 3:** Quality of FOF intervention evidence.

Interventions		Relative effect (95% Cl)	*p*-value	Studies (participants)	a	b	c	d	e	Quality of the evidence
The overall effect of interventions on FOF										
Racey et al., 2021 (21)		−0.73 (−1.10,-0.36)	*p* = 0.114	8 (263)	−1^1)^	0	0	0	0	Moderate
Kruisbrink et al., 2022 (22)		−0.36 (−0.48,-0.25)	*p*<0.001	52 (None)	−1^1)^	−1^2)^	0	0	0	Low
Zijlstra et al., 2007 (23)		—	—	19 (3067)	−1^1)^	−1^2)^	0	0	0	Low
Cognitive behavior therapy (CBT)										
Chua et al., 2019 (17)	Immediate	−0.28 (−0.35, −0.21)	*p* < 0.0001	15 (3165)	−1^1)^	0	0	0	0	Moderate
	≤ 6 months	−0.32 (−0.49, −0.15)	*p* = 0.003	6 (3160)	−1^1)^	−1^2)^	0	0	0	Low
	>6 months	−0.30 (−0.45, −0.14)	*p* = 0.0002	4 (1403)	−1^1)^	−1^2)^	0	0	0	Low
Papadimitriou et al., 2020 (36)	2–5 months	−0.30 (−0.50, −0.10)	*p*<0.003	5 (None)	−1^1)^	−1^2)^	0	0	0	Low
	6–12 months	−0.29 (−0.49, −0.09)	*p*<0.005	5 (None)	−1^1)^	−1^2)^	0	0	0	Low
Liu et al., 2018 (37)	Immediate	−0.33 (0.21, 0.46)	*p*<0.001	6 (1626)	0	0	0	0	0	High
	>12 months	−0.37 (0.21, 0.53)	*p*<0.001	6 (1626)	0	0	0	0	0	High
Physical-activity therapy (PAT)										
Savvakis et al., 2024 (24)		—	—	10 (None)	−1^1)^	0	0	0	0	Moderate
Kumar et al., 2016 (26)		0.37 (0.18, 0.56)	*p* = 0.0001	30 (2878)	−1^1)^	−1^2)^	0	0	0	Low
Feng et al., 2022 (27)		−0.34 (−0.44, −0.23)	*p*<0.001	12 (547)	−1^1)^	0	0	0	0	Moderate
Kendrick et al., 2014 (25)	Immediate	0.17 (−0.06, 0.39)	*p* = 0.14	4 (380)	−1^1)^	0	0	−1^3)^	0	Low
	<6 months	0.17 (−0.05, 0.38)	*p* = 0.12	4 (356)	−1^1)^	0	0	−1^3)^	0	Low
	>6 months	0.20 (−0.01, 0.41)	*p* = 0.68	3 (386)	−1^1)^	0	0	−1^3)^	0	Low
Low-intensity PAT intervention										
Feng et al., 2022 (27)	Balance exercise	−0.62 (−0.93, −0.31)	*p* = 0.002	12 (547)	−1^1)^	−1^2)^	0	0	−1^4)^	Very low
	Resistance exercise	−0.04 (−0.26, 018)	*p* = 0.17	4 (1147)	−1^1)^	0	0	0	−1^4)^	Very low
Moderate-intensity PAT intervention										
Weber et al., 2020 (28)	Tai Chi/Qigong	0.79 (0.33, 1.26)	*p*<0.001	7 (None)	−1^1)^	−1^2)^	0	−1^3)^	0	Very low
	yoga Pilates	0.58 (0.23, 0.93)	*p* = 0.007	4 (None)	−1^1)^	−1^2)^	0	−1^3)^	0	Very low
	Total	0.71 (0.39, 1.03)	*p* = 0.0002	37 (3224)	−1^1)^	0	0	−1^3)^	0	Low
Logghe et al., 2010 (29)	Tai Chi	0.27 (−0.18, 0.72)	—	2 (None)	−1^1)^	0	0	−1^3)^	−1^4)^	Very low
Silva et al., 2021 (30)	Pilates	−0.02 (−0.37, 0.33)	*p* = 0.91	5 (225)	−1^1)^	0	0	−1^3)^	0	Low
High-intensity PAT intervention										
Veronese et al., 2017 (31)	Dance	—	—	10 (680)	−1^1)^	0	0	0	0	Moderate
Silva et al., 2018 (32)	Dance	−8.61 (10.16, −7.07)	*p*<0.0001	5 (225)	−1^1)^	−1^2)^	0	−1^3)^	−1^4)^	Very low
Ge et al., 2022 (33)	Exergames	—	—	23 (2083)	−1^1)^	0	0	0	0	Moderate
Melo et al., 2023 (34)	The aquatic physical exercises	—	—	2 (66)	−1^1)^	0	0	0	0	Moderate
Papalia et al., 2020 (35)	Land-based or aquatic exercise	−0.13 (−0.28, 0.03)	*p* = 0.10	4 (858)	−1^1)^	0	0	0	0	Moderate
Feng et al., 2022 (27)	Aerobic	−0.41 (−1.16, 0.33)	*p* = 0.09	2 (114)	−1^1)^	−1^2)^	0	−1^3)^	−1^4)^	Very low
	3D exercise	−0.36 (−0.56, −0.16)	*p* = 0.002	7 (1579)	−1^1)^	−1^2)^	0	0	−1^4)^	Very low

a, Limitations in design; b, Inconsistency; c, Indirectness; d, Imprecision; e: Publication bias.

1) There are defects in randomness, concealment, blinding, etc.; 2) The *I*^2^ >50% and the heterogeneity is higher; 3) The sample size is small or the confidence interval is wide; 4) Publication bias has not been assessed or may be significant.

“—” indicates that no meta-analysis was performed or reported in the literature.

### 3.5 Effect of interventions on FOF

Three reviews were conducted to analyze and report on multi-component interventions for FOF systematically. According to Zijlstra et al. (23), 11 out of 19 preventive measures were found to decrease FOF, including Tai Chi (*n* = 3), exercise interventions (*n* = 2), fall-related multifactorial programs (*n* = 5), and hip protective interventions (*n* = 1). In a meta-analysis involving a large sample, it was found that combining PAT with CBT led to a significant reduction in FOF [SMD = −0.36, 95% CI (−0.48, −0.25), *p* < 0.0001] at the initial available assessment following the intervention (22). Furthermore, univariate meta-regression showed that interventions incorporating meditation, holistic exercise (such as Tai Chi or Pilates), or body awareness were significantly more effective than those without these components. Additionally, Racey et al. (21) evaluated the effectiveness of fall prevention interventions for community-dwelling adults with mild to moderate cognitive impairment. The results showed that PAT interventions are effective at improving FOF [SMD = −0.73, 95% CI (−1.10, −0.36), *p* < 0.05]. However, high-quality studies with longer follow-ups and adequate sample sizes are needed to determine their direct effectiveness on FOF.

#### 3.5.1 PAT intervention on FOF

PAT is available in various formats and classified as low, moderate, and high-intensity based on global recommendations (38). Low-intensity PAT typically involves balance or resistance training; moderate-intensity PAT primarily comprises Tai Chi, Qigong, Yoga, Pilates, and other mind–body interventions; and high-intensity PAT can be land-based or aquatic, such as dance, exercise games, swimming, or other holistic exercises. The total impact of PAT on FOF was reported in two studies. Savvakis et al. (24) found that PAT interventions were associated with a small to moderate reduction in FOF immediately post-intervention [SMD = 0.37, 95% CI (0.18, 0.56), *p* < 0.0001], which was consistent with the findings of Feng et al. (27). Considering the susceptibility of older adults, we classified all PAT interventions as low, moderate, or high intensity based on the nature of the activities. The findings of the subgroup are as follows:

- *Low-intensity PAT on FOF: Low*-intensity PAT interventions include balance and resistance training. Feng’s study (27) found that balance PAT intervention had the most significant effect size [SMD = −0.62, 95% CI (−0.93, −0.31), *p* < 0.001], compared to studies using resistance PAT, aerobic PAT, or other forms of interventions, all of which also showed significant results.
- *Moderate-intensity PAT on FOF:* Three reviews reported the effects of moderate-intensity PAT, such as Tai Chi, Qigong, and Yoga/Pilates on FOF, with differing outcomes. In the study conducted by Weber et al. (28), moderate-intensity PAT interventions significantly reduced FOF compared to a non-PAT control group [SMD = 0.12, 95% CI (−0.16, −0.39), *p* = 0.08]. Similarly, Silva et al. (30) showed that the Pilates group had a lower FOF score, with statistically significant differences from the control group [MD = −8.61, 95% CI (−10.16, −7.07), *p* < 0.001]. However, a small sample meta-analysis suggested that moderate-intensity PAT for community-dwelling people with FOF did not show any improvement [SMD = 0.27, 95% CI (−0.18, 0.72), *p* > 0.05] compared to non-PAT controls (29).
- *High-intensity PAT on FOF:* Six studies reported the effect of high-intensity PAT on FOF. Silva et al. (32) demonstrated that technology-mediated dance systems showed little or no difference from the control groups [SMD = −0.02, 95% CI (−0.37, −0.33), *p* = 0.91]. A systematic review indicated that 15 of 23 trials (65%) involving exergame intervention, which mixes interactive features of video games with exercise, were associated with a statistically significant reduction in FOF (33). Another review yielded inconclusive results; however, two primary randomized controlled trials (RCTs) suggested that dancing may reduce FOF compared to the control group (31). Additionally, a systematic review indicated that aquatic PAT was more effective than land-based PAT in reducing FOF (34). In contrast, another meta-analysis concluded that land-based exercise interventions were ineffective relative to the control group [SMD = −0.13, 95% CI (−0.28, 0.03), *p* = 0.10] (35).

**Figure 2:**
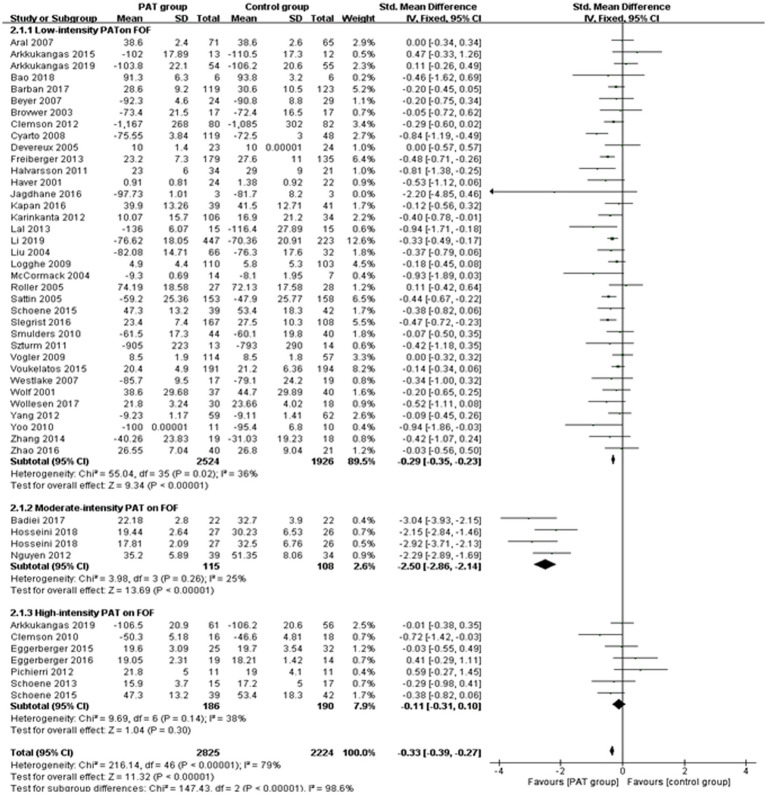
Forest plots of the low-intensity, moderate-intensity, and high-intensity PA T on FOF.

A supplementary meta-analysis of the principal RCT papers was performed. The results showed that both low-intensity PAT [SMD = −0.28, 95% CI (−0.37, −0.20), *p* < 0.0001] and moderate-intensity PAT [SMD = −2.52, 95% CI (−2.94, −2.11), *p* < 0.0001] can effectively reduce FOF incidence, while high-intensity PAT [SMD = −0.10, 95% CI (−0.37, 0.17), *p* = 0.48] was not significant. The comprehensive subgroup data are presented below, and the forest plots of the low-intensity, moderate-intensity, and high-intensity PAT on FOF are illustrated in Figure 2.

#### 3.5.2 CBT intervention on FOF

The impact of CBT on FOF was reported in four studies. Chua et al. (17) found that CBT was effective in reducing FOF immediately post-intervention [SMD = −0.28, 95% CI (−0.35, −0.21), *p* < 0.0001], as well as in the short term (<6 months) [SMD = −0.32, 95% CI (−0.49, −0.15), *p* = 0.003], and in the long term (≥6 months) [SMD = −0.30, 95% CI (−0.45, −0.14), *p* = 0.002]. Papadimitriou et al. (36) consistently reported that CBT interventions were effective in reducing FOF in both short-term (2–5 months) [SMD = −0.30, 95% CI (−0.50, −0.10), *p* < 0.003] and long-term follow-ups (6–12 months) [SMD = −0.29, 95% CI (−0.49, −0.09), *p* < 0.005]. Similarly, Liu et al. (37) indicated that CBT interventions have substantial immediate [SMD = −0.33, 95% CI (0.21, 0.46), *p* < 0.001] and retention [SMD = −0.37, 95% CI (0.21, 0.53), *p* < 0.001] effects on reducing FOF for up to 12 months.

**Figure 3:**
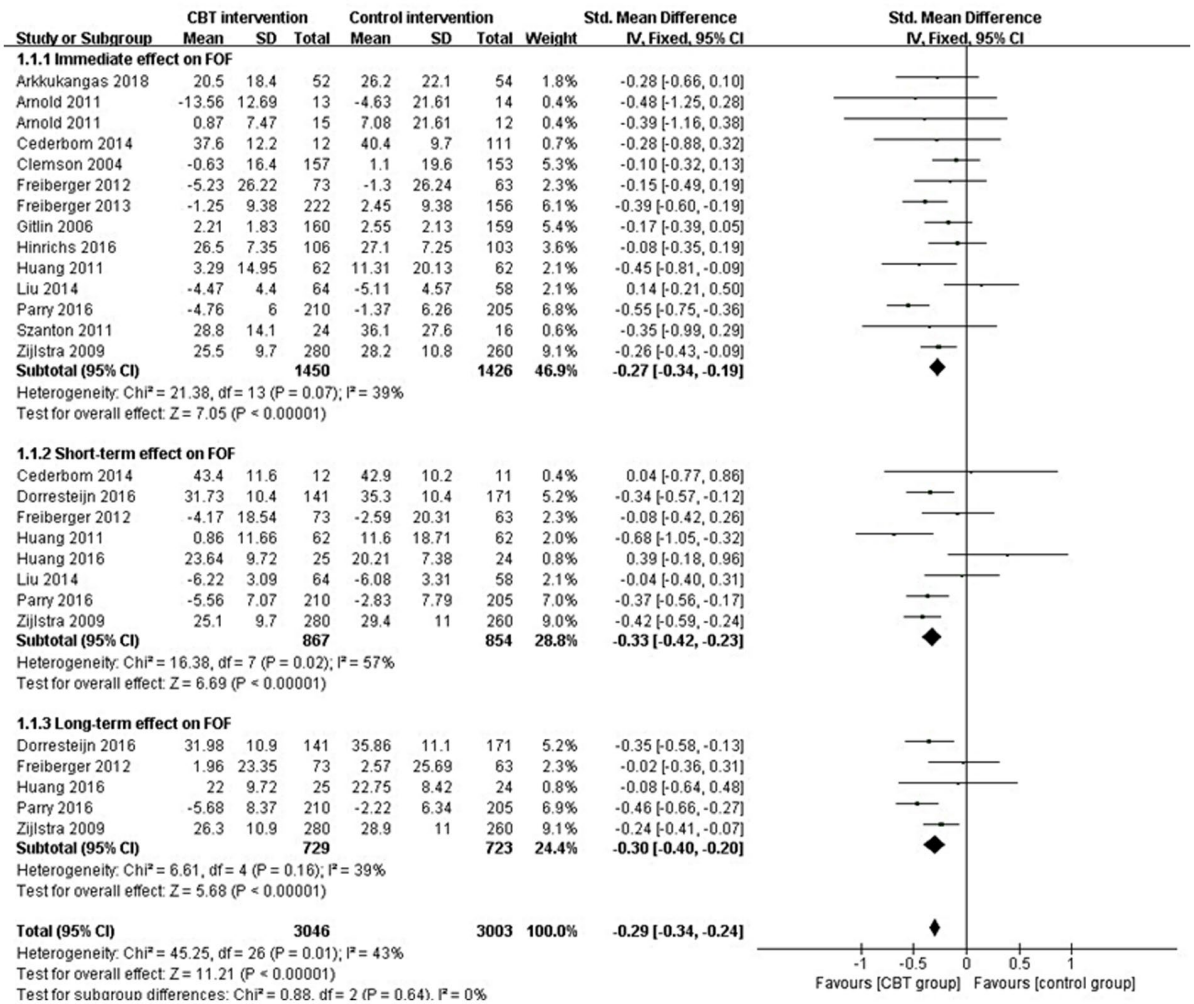
Forest plots of the immediate (1 month), short-term (<6 months), and long-term (>6 month) effects of CBT on FOF.

A supplementary meta-analysis was conducted, which included primary RCT evidence. The findings indicated that CBT can effectively decrease FOF [SMD = −0.30, 95% CI (−0.35, −0.25), *p* < 0.001] for at least 1 year, with its impact attaining the peaking in a short-term (<6 months). Details of the subgroup results are presented in Figure 3.

### 3.6 Publication bias

**Figure 4:**
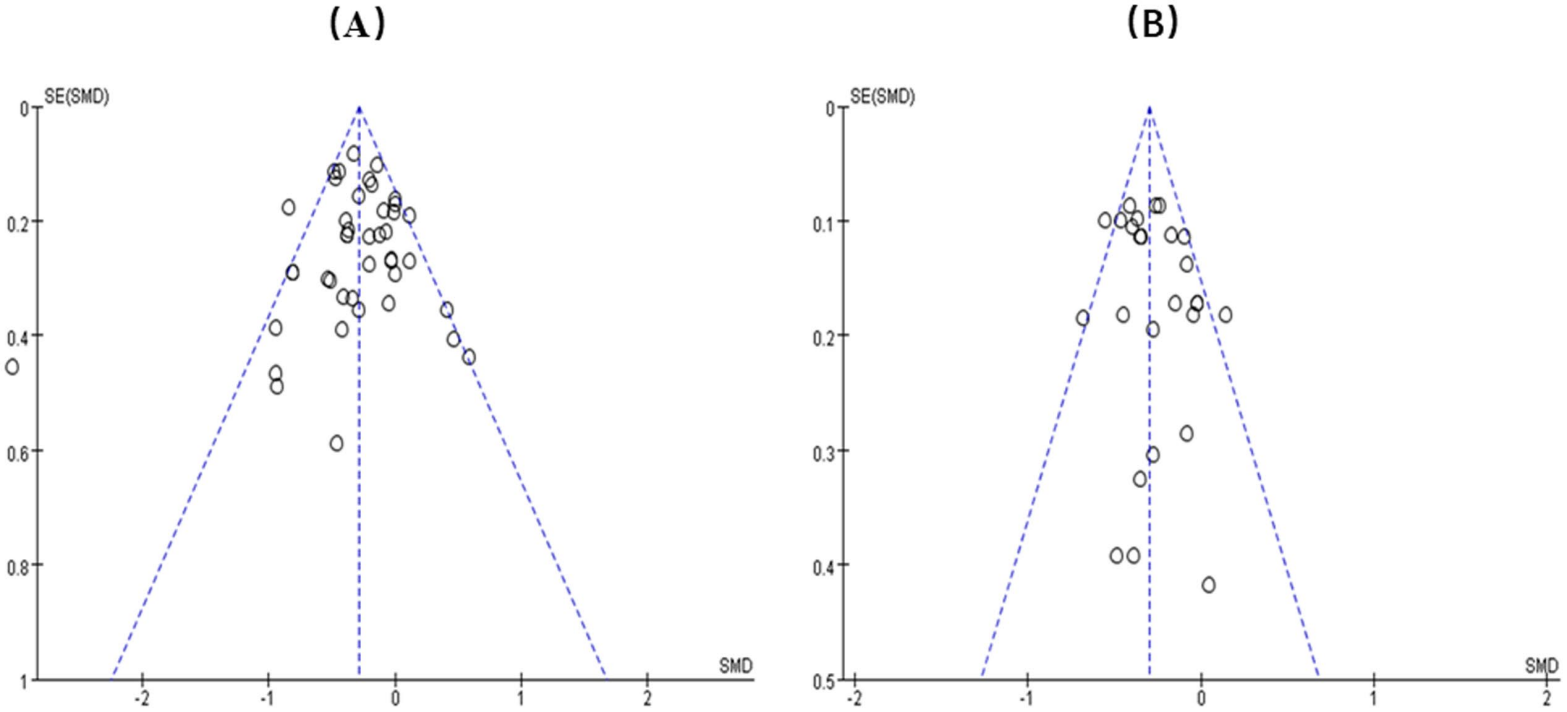
Funnel plots of the effects of PAT **(A)** CBT **(B)** on FOF.

Funnel plots were employed to detect publication bias in the PAT and CBT intervention groups. The findings are illustrated in Figure 4. The funnel plots seemed symmetrical, showing minimal heterogeneity among the trials.

## 4 Discussion

### 4.1 Main findings

This umbrella review aimed to systematically overview the impacts of PAT and CBT interventions in reducing FOF among older adults. Based on the synthesis of the 18 included reviews, the majority focused on PAT interventions (*n* = 12), three papers mainly discussed CBT interventions, and three explored the overall effect of multi-component FOF interventions. Clear evidence supports the conclusion that a comprehensive intervention strategy that addresses physical and psychological factors is more effective than isolated approaches to managing FOF. Our supplementary meta-analysis indicates that low-to moderate-intensity PAT is more suitable for reducing FOF in older adults, and CBT effects may persist for up to 1 year.

### 4.2 Quality of the evidence

The GRADE evaluation revealed significant limitations in the quality of evidence. Of the 31 pieces of evidence assessed, two were classified as high quality, eight as moderate quality, 12 as low quality, and nine as very low quality. Firstly, most studies had defects in randomness, concealment, and blinding, leading to design limitations. Secondly, the inconsistency was primarily due to the heterogeneity among trials, stemming from variations in the intervention measures’ frequency, duration, and intensity. PAT interventions were classified into three subgroups according to exercise intensity: low-intensity, moderate-intensity, and high-intensity, with *I*^2^ values of 36, 25, and 38%, respectively, demonstrating no substantial heterogeneity within the subgroups. When combined, the *I*^2^ value was 79%, indicating that intervention intensity might be a key factor contributing to heterogeneity, confirming our analysis. Furthermore, the small sample sizes resulted in broad confidence intervals, which introduced imprecision and lowered the quality of evidence. Issues such as incomplete systematic review/meta-analysis retrieval, small sample sizes, and lacking funnel plots contributed to publication bias. Future studies should focus on designing robust research methodologies to provide stronger evidence for clinical practice.

### 4.3 Agreements and disagreements with other studies or reviews

The intensity of PAT intervention plays a crucial role in reducing FOF among older adults. Our main finding is that low-to-moderate-intensity PAT programs are particularly effective in reducing the FOF. One of the key advantages of low-to-moderate-intensity exercise programs is their accessibility and sustainability for older adults. For instance, a randomized controlled trial demonstrated that community-based Baduanjin exercise intervention for older adults is a safe, feasible, and acceptable exercise program that can be effective in alleviating vital exhaustion reduce FOF (39). Secondly, such programs are often more cost-effective and accessible than high-intensity alternatives, making them suitable for a broader range of participants, including those with varying fitness and health conditions (40). Moreover, psychological benefits also play an important role in these low-to moderate-intensity programs. For instance, group exercise settings such as Tai Chi can foster a sense of community and support, enhancing motivation and adherence, which enhances the positive effects on both physical and mental health (41).

Conversely, evidence suggests that high-intensity PAT may not effectively reduce FOF in older adults. Firstly, the physical condition of older adults is an important factor in their response to exercise. Lin et al. (42) emphasize that the type, intensity, and duration of exercise must be tailored to the individual’s physical condition to prevent falls effectively. Therefore, high-intensity PAT may not be suitable for those with pre-existing conditions or low baseline fitness, as it may lead to injury or falls during activity. Similarly, Barreto et al. (43) suggest that the notion of “more exercise is always better” does not apply universally to vulnerable older adults, as excessive exercise may lead to overtraining, diminished immunity, and negative psychological outcomes. In line with these findings, a systematic review indicated that traditional moderate or high-intensity physical resistance training alone did not significantly reduce the risk of falls (44). This suggests that while high-intensity PAT may improve specific physical capacities, it does not adequately address the multifaceted nature of fall prevention, including balance and functional training. Instead, focusing on low-to moderate-intensity PAT while incorporating balance and functional training is more beneficial for this population.

The study also identified CBT as a significant intervention for addressing FOF in older adults. However, more research is needed to determine whether regular intensive therapy is necessary to maintain treatment effects, as reflected in a recent systematic review (45). On one hand, CBT helps older adults reframe their thoughts about falling, viewing it as a manageable risk rather than an inevitable outcome, which significantly reduces anxiety and promotes engagement in physical activities (46). Besides, by addressing these psychological factors, CBT reduces FOF and promotes a more active and engaged lifestyle among older adults (33). On the other hand, whether CBT requires long-term continuous intervention is nuanced and may depend on individual circumstances and the complexity of the issues being addressed. For example, the systematic review by Jönsson et al. (47) supports the notion that psychological treatments, including CBT, can lead to long-term improvements in mental health outcomes for older adults, particularly in reducing depressive symptoms. Conversely, Kim et al. (48) highlighted that interventions lasting only 8 weeks might not affect lasting behavioral changes in older adults. Similarly, Lenouvel et al. (45) found that CBT with or without exercise interventions for FoF probably sustains improvements beyond 6 months, implying that addressing these underlying issues through initial intensive therapy may yield lasting benefits.

The Global Initiative World Guidelines recommend a multidisciplinary approach that includes PAT, CBT, and occupational therapy to reduce FOF among older adults (1B) (49). These findings suggest that integrating low-to-moderate PAT with CBT interventions provides a holistic approach to managing FOF. For example, a randomized controlled trial emphasized the importance of combining CBT with Tai Chi exercise to enhance mobility, manage FOF, and improve the quality of life among community-dwelling older adults (50). Moreover, Wetherell et al. (51) developed the ABLE intervention, which combines exposure therapy, cognitive restructuring and physical activity, demonstrating that such integrative approaches can significantly reduce FOF in older adults. Similarly, Yoshikawa et al. (52) developed the Matter of Balance Volunteer Lay Leader (AMOB/VLL) model by combining CBT with strength and balance exercises. In summary, Interventions combining PAT with CBT yield better outcomes in reducing FOF than PAT alone, as they tackle mental and physical barriers to activity. Future research should continue to explore the synergistic effects of these interventions to optimize fall prevention strategies for older populations.

### 4.4 Limitations and implications

This umbrella review has several limitations that should be noted. First, there is limited available evidence regarding the long-term effectiveness of CBT interventions for mitigating FOF. Although we have strengthened the theoretical basis for its effectiveness through its potential mechanisms, drawing definitive conclusions about its long-term impact remains challenging. Similarly, it remains unclear whether PAT effects are sustained over time, and limited data make direct comparisons between PAT and CBT interventions unfeasible. Nevertheless, it is clear that psychological improvements play a crucial role in reducing FOF, and combining PAT with CBT could offer a more comprehensive approach to managing this condition in older adults. Secondly, the FOF assessment tools for older adults have not been standardized; although the SMD effect size was used, the pooled effect size did not differ significantly among the various scales. Further, the results may have been affected by a high risk of performance bias due to the infeasibility of blinding participants and therapists, given the nature of the intervention.

Despite some limitations, several practical implications can be drawn from this study. Firstly, PAT serves as a foundational component in reducing FOF, as it directly addresses the physiological factors contributing to fall risk. CBT focuses on cognitive restructuring, helping individuals develop more realistic perceptions of their fall risk and enhancing their confidence in their physical abilities. Our study supports that a comprehensive intervention strategy is more effective than isolated approaches, as it addresses the multifaceted nature of fear and fall risk. Secondly, PAT programs should be flexible and adaptable. Low-to-moderate-intensity exercises are especially beneficial for frail individuals, while high-intensity programs may provide additional benefits regarding strength and balance improvement. Therefore, tailoring exercise interventions to the individual’s physical capabilities and fear levels is essential for maximizing their effectiveness in reducing FOF. Furthermore, CBT profoundly influences FOF in older adults by altering cognitive perceptions, enhancing coping strategies, and improving overall mental health. Integrating CBT with educational programs provides a comprehensive approach to managing FOF, ultimately promoting better health outcomes and quality of life for older adults. However, this psychological condition is not merely a transient concern; it often necessitates ongoing intervention to effectively manage and mitigate its effects on FOF, particularly in cognitively impaired older adults.

## 5 Conclusion

The umbrella review found that PAT and CBT interventions can effectively address FOF in older adults. While regular intensive CBT may not be universally necessary for all older adults with FOF, ongoing engagement in CBT and low-to moderate-intensity PAT interventions provide a comprehensive strategy for managing FOF and improving overall quality of life. Potential directions for future research include tracking the long-term effects of comprehensive intervention programs. Finally, we hope this study can provide valuable references for FOF intervention theory and practice while stimulating further discussions and research.

## Data Availability

The original contributions presented in the study are included in the article/supplementary material, further inquiries can be directed to the corresponding authors.
